# Wolves and dogs recruit human partners in the cooperative string-pulling task

**DOI:** 10.1038/s41598-019-53632-1

**Published:** 2019-11-26

**Authors:** Friederike Range, Alexandra Kassis, Michael Taborsky, Mónica Boada, Sarah Marshall-Pescini

**Affiliations:** 1 Domestication Lab, Konrad Lorenz Institute of Ethology, University of Veterinary Medicine, Vienna, Savoyenstraße 1a, A-1160 Vienna, Austria; 2 Comparative Cognition, Messerli Research Institute, University of Veterinary Medicine, Vienna, Medical University of Vienna, University of Vienna, Vienna, Austria; 3 Division of Behavioural Ecology, Institute of Ecology and Evolution, University of Bern, 3032 Hinterkappelen, Switzerland; 4 Grupo UCM de Psicobiología Social, Evolutiva y Comparada, Departamento de Psicobiología, Facultad de Psicología, Campus de Somosaguas, Universidad Complutense de Madrid, 28223 Madrid, Spain

**Keywords:** Evolutionary developmental biology, Animal behaviour

## Abstract

In comparison to non-human animals, humans are highly flexible in cooperative tasks, which may be a result of their ability to understand a partner’s role in such interactions. Here, we tested if wolves and dogs could flexibly adjust their behaviour according to whether they needed a partner to solve a cooperative loose string-pulling paradigm. First, we presented animals with a delay condition where a human partner was released after the subject so that the animal had to delay pulling the string to enable coordinated pulling with the human partner. Subsequently, we investigated whether subjects would recruit a partner depending on whether they could operate the apparatus alone, or help from a partner was required. Both wolves and dogs successfully waited in the delay condition in 88% of the trials. Experimental subjects were also successful in recruiting a partner, which occurred significantly more often in the cooperation trials than in the solo pulling condition. No species differences were found in either experiment. These results suggest that both wolves and dogs have some understanding of whether a social partner is needed to accomplish a task, which enables behavioural coordination and cooperation.

## Introduction

Cooperation can be defined as two or more individuals acting together to reach a common goal^1^. It is widely observed in animals, including humans^2–4^. Human cooperation is characterized by enormous flexibility in response to one another, which may be explained by the fact that humans can recognize the role of a social partner in cooperative interactions and adjust their behaviour accordingly^5^.

When testing such understanding in non-human animals, the loosestring- pulling paradigm has often been used. In this task, two individuals have to simultaneously pull on two ends of a rope to bring a baited apparatus within reach^6^; both individuals must coordinate their actions to pull at the same time, otherwise the rope will come loose and the apparatus becomes non-functional. To test whether animals link the presence of a partner to the cooperative action, one can delay the release of one partner so that the test subject has to wait for the partner to arrive at the apparatus before starting to pull the rope^7^. A number of species have been shown to wait for the partner in such conditions (chimpanzees^7^, wolves^8^, elephants^9^, keas^10^, bottlenose dolphins^11^) whereas others do not (ravens^12^, but see^13^; grey parrots^14^; rooks^15^).

The behaviour of animals exposed to such challenges can also be compared between a cooperative and a solo condition to test if they understand the role of the partner. While in the cooperative condition, the subject needs a partner to solve the task, in the solo condition the string-pulling apparatus is altered so that animals can solve the task by themselves. Different experimental designs revealed that various animal species show some sort of understanding of such tasks (e.g. chimpanzees^16^, elephants^9^, grey parrots and keas^10,14^, but see rooks^15^).

An important limitation of these studies, except the one in chimpanzees^16^ and another one about interspecific cooperation between groupers and morey eels^17^, is that the animals did not need to actively recruit a partner (for example by opening a door for them). Recruiting a partner is arguably more challenging than choosing the cooperative apparatus when the partner is already present and the single apparatus when alone (keas, grey parrots), or inhibiting pulling to wait for a partner to be released to solve the task (elephants^9^). Notably, recruiting a partner requires the test subject to move away from the visibly baited apparatus to operate the partner release mechanism. Moving away from a food source to perform another action has been shown to be challenging for many species since it requires considerable inhibitory control (e.g.^18–22^). Furthermore, when the test subject inspects the apparatus, it has to make a decision, in the absence of a partner, whether to recruit or not. Considering their extensive previous experience with the apparatus in all of these studies, animals may have acquired a simple rule of thumb: “if the partner is present, go to the cooperative apparatus”. This rule, however, is insufficient when the partner has to be actively recruited.

Here we tested if wolves and dogs understand the need for a partner in the loose string-pulling paradigm. We previously tested these same dogs and wolves, which were raised and kept under similar conditions, in two studies involving conspecific and human partners whose release was delayed or simultaneous. While wolves were overall successful in waiting for their partner for up to 10 seconds in the delay condition^8^, dogs failed already when their conspecific partner was released simultaneously (3%), despite extensive training in the task^8^. However, this failure was apparently mainly due to the lack of tolerance towards the partner^8,23^. Indeed, when tested with a human partner in the simultaneous release condition, the dogs succeeded much more often than with the conspecific partner (49% of the trials with human; although still at chance level), while the wolves performed significantly above chance (61.5%); however, statistically, dogs and wolves did not differ from each other^24^.

In a related study^25^, pet dogs were tested both with conspecifics and with their owner in several conditions including a partner delay condition. While the pet dogs were successful with both conspecific and human partners when they were released at the same time, in the delay condition, in which the partners were slowed down after the simultaneous release by forcing them to pass through a maze of obstacles, dogs waited for their conspecific but not for the human partner. This may be explained by the fact that the human delay ($\bar{{\rm{x}}}$ = 15.6 seconds) was longer than the conspecific delay ($\bar{{\rm{x}}}$ = 2.2 seconds). These results call into question whether dogs recognize the need for a partner as the delay with the conspecific was so short that it hardly differed from the condition, in which they were not delayed, and only two of seven dogs delayed their pulling for more than 2 seconds.

In the current study, we aimed to directly compare the wolves’ and dogs’ understanding of the need for a partner in the string-pulling task. We did this by testing 1) whether test subjects would wait for a human cooperation partner in a delay condition (experiment 1), and 2) whether they would adjust their recruitment of a human partner to the contingencies of the task (solo vs. cooperative pulling, experiment 2).

## Experiment 1: Delay Condition

### Methods

All animals that were tested in this experiment had a history of operating the apparatus and had reached a similar level of success^24^. Accordingly, they had ample experience with the entire setup.

**Figure 1:**
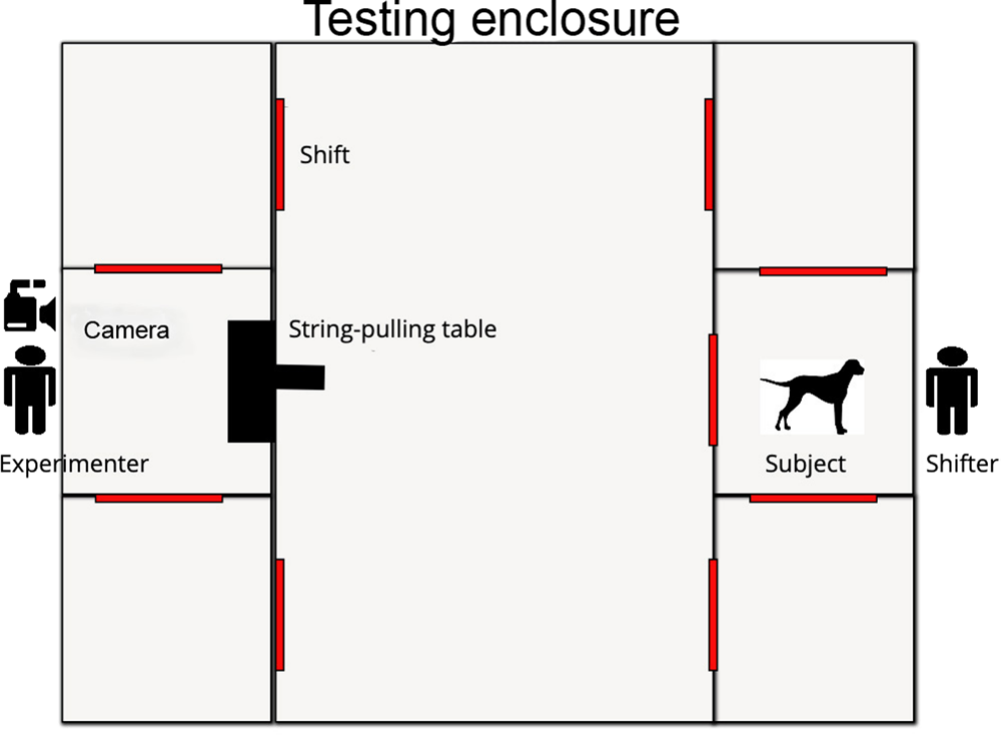
**S**et-up of the test.

A total of 17 animals were tested; 9 wolves (7 males, 2 females; mean age: 7.1 years, Table S1) and 8 mixed-breed dogs (5 males, 3 females; mean age: 5.2 years, Table S1) that were raised and kept under comparable conditions (see Supplementary Information). The human partners were professional animal trainers, who have worked with the wolves and dogs at the centre on a daily basis. We used a string-pulling apparatus comparable to the ones used in previous studies (e.g.^9,23^) (Supplementary Information, Fig. S1). The tests were conducted in outside test enclosures equipped with two shifting systems on opposing sides of each enclosure. Each shifting system was separated into 3 compartments, interconnected to each other and connected with the enclosure by multiple sliding doors (Fig. 1). After a 5-minute exploration phase, the test subject was shifted into the middle compartment of the shifting system (approximately 25 m from the apparatus), where the human partner already waited. The human greeted the animal for approximately one minute before she moved either to the adjacent left or right shifting compartment (sequence randomized within and across animals). After the experimenter entered the test enclosure and prepared the apparatus, she stepped in front of the apparatus facing the subject and human cooperation partner, called their names, and showed them the two rewards (a piece of sausage in each hand; brand “Geiger”, type “Knacker”) to get the subjects’ attention, before placing them on the wooden trays. Afterwards, the experimenter left the test enclosure and the subject was released by the helper. The partner was released 10 seconds later. For details of procedure and experimental rules please see the Supplementary Information. In general, the human partner was instructed not to communicate with the animal and once touching the rope, either to pull in unison with the animal or pull no matter what after 10 seconds. All human partners adhered to the set rules of how to behave during the experiment.

A trial lasted until either the dyad had successfully solved the task, or the subject or the human had pulled out the rope. Then the subject and the human were called back into the middle compartment to start the next trial. For the behavioural analysis, all experiments were videotaped using a camcorder that was placed behind the apparatus. A total of two sessions, conducted on different days, each consisting of six trials, were carried out for each test subject. A success was defined as the animal obtaining the food by operating the apparatus together with the human partner. Twenty percent of the data were coded by a second rater. Interobserver accordance (calculated with Cohen’s kappa) for the success was at 100%. We used Generalized linear mixed models (with a binomial distribution) with success as the dependent variable, species and session as explanatory variables, and subject identity as a random factor. The full model (with all explanatory factors) was compared to a null model with subject identity as a random effect. All statistical computations were performed with R (version 3.4.1), package lme4^26^.

## Results and Discussion

Animals were successful on average in 5.3 (±1.4) trials per session (range 0–6). Except for one wolf (Amarok) that succeeded in only one trial in the first session and none in the second session, and one dog (Meru) that succeeded in only 7 out of 12 trials (across the two sessions), all other animals were successful in at least 10 out of 12 trials (see Table 1). The full model did not significantly differ from the null model (χ2 = 0.1597 p = 0.923), suggesting success was affected by neither species nor session.

**Table 1:** Percentage of successful trials across the two sessions in the delay condition.

Individual	Species	% Success	Individual	Species	% Success
Chitto	Wolf	100	Sahibu	Dog	91.7
Amarok	Wolf	8.3	Nuru	Dog	91.7
Tala	Wolf	91.7	Binti	Dog	100
Geronimo	Wolf	100	Nia	Dog	91.7
Kenai	Wolf	91.7	Meru	Dog	58.3
Nanuk	Wolf	100	Pepeo	Dog	100
Aragorn	Wolf	91.7	Asali	Dog	91.7
Yukon	Wolf	83.3	Panya	Dog	100
Kaspar	Wolf	100			

Both dogs and wolves were highly successful in the delay condition, on average waiting for their partner in 88% of the trials. We found no difference between the wolves’ and dogs’ performance in the delay condition, suggesting that their understanding of the task may be similar. However, since previous studies have suggested that dogs show a greater propensity to stay in proximity to humans than wolves^27^, it could still be that the success of dogs in the delay condition was due to their motivation to stay close to the human, rather than an understanding of the need for a partner to solve the task. We therefore tested the wolves’ and dogs’ understanding of the task further, by presenting a solo vs. cooperative apparatus and assessing whether animals would ‘recruit’ their human partner only when needed.

## Experiment 2a: Cooperative vs. Solo Condition

### Methods

The aim of the second experiment was to investigate if wolves and dogs recruit a partner only when needed. Accordingly, we performed sessions consisting of 4 solo trials, in which the animals could solve the task alone, and 4 cooperation trials, in which they needed to recruit a partner in order to solve the task and obtain the food reward. Before testing, all animals experienced that if only one rope was attached to the middle of the apparatus, they could solve the task alone (see Supplementary Information, Fig. S2). Furthermore, we trained the animals to step on a marker (a wooden star-shaped board placed on the ground), which was paired with the opening of a sliding door that gave access to a small compartment (see Supplementary Information). In the test session, by stepping on one of two markers, that was adjacent to the partner waiting in the small compartment, the animals could open the sliding door for the human partner, who then joined them at the apparatus.

Success was defined as obtaining the food reward with the partner’s help in the cooperation trials, and alone in solo trials. However, because we were interested in how animals reached their success (hence how they discriminated between cooperation and solo trials), additional behaviours were analysed. The animals could potentially distinguish between the solo vs. cooperation trials by (1) the baiting procedure, where in the solo condition only the animal was called by name and only a single treat was shown compared to the cooperative condition, in which both the animal and the human were called by name and two treats were shown; (2) visiting the table and just looking at the setup with a centrally placed rope in the solo trials and the two ends far apart in the cooperative condition or (3) visiting the table and starting to pull the rope to receive some sensory feedback (i.e. does the rope give way, or does the pulling affect the movement of the tray). Hence, we analysed the likelihood that animals would (1) step on the correct marker before visiting the table (suggesting they either used the human baiting procedure to discriminate between trial types or just stepped on the marker, because they were trained to do so); (2) approach the table before stepping on the marker (suggesting they required some inspection of the table setup to discriminate between trial types); and (3) pull the rope once they were at the table (indicating that the assessment was based also on direct sensory feedback when manipulating the rope).

If the animals merely pulled the string every time, as trained, they would be successful in all solo trials, but would not succeed in the cooperative trials, predicting a success rate of 50%. Accordingly, we predicted that if animals understood the task, their success rate would be above 50%, which would indicate their capacity to recruit the human partner at least sometimes, when this was necessary. We further predicted that, given the additional steps required to succeed in the cooperative condition as well as the need for inhibitory control abilities known to be taxing for these species^20,25,28^, subjects should be significantly more successful in solo vs. cooperative condition.

Moreover, since we did not expect the animals to pick up on the differences in the baiting procedure, we predicted that they would first visit the table and also potentially try pulling on the rope to obtain information about the ‘trial type’ rather than stepping on the marker as their first action after release. Furthermore, if their understanding is based on the visual inspection of the table and they understand that a partner is needed in the cooperation trials, we predicted that they would pull the rope more often in solo than in cooperation conditions and that they would step on the correct marker more often in cooperation than in solo trials. For all these variables, we predicted a learning effect, whereby animals would perform better in the later sessions. Considering that wolves may have a better causal understanding than dogs^29–31^, we predicted that overall wolves would show a better understanding of the task, and hence a higher success rate, than dogs.

8 wolves (6 males and 2 females, ranging from 4 to 8 years of age, mean age: 7.3 years, Table S1) and 7 mixed-breed dogs (5 males and 2 females, ranging from 2 to 7 years of age, mean age: 5.5 years, Table S1), that had been successful in the delay condition, were tested in the second experiment.

Six sessions were conducted on separate days, each including 4 solo and 4 cooperation trials in a semi-random order with no more than two consecutive trials of the same type. After a 1 minute greeting in the middle compartment, the human cooperation partner was shifted to the left or right compartment (pseudo randomized), addressing the animal after entering the compartment to ensure that the animal paid attention to where the human was positioned (see Fig. 1). Next, the experimenter entered the test enclosure and placed one marker each next to the sliding doors of the left and right shifting compartment adjacent to the subject’s compartment. While doing so, the experimenter called the animal’s attention, waiting until it oriented towards the markers and holding them up for a second. Next, the experimenter walked back to the string-pulling apparatus and lifted both arms waving a food reward in each hand while calling the animal. The experimenter waited until the subject oriented towards her to place the sausage on the board, and subsequently left the test enclosure. Then the animal was released. The procedure was identical in the solo condition, with the exception that only one arm was raised with the respective piece of food, which was then placed on the apparatus, and only the animal (and not the partner) was called by name.

The animal was allowed two minutes to step on the marker or to obtain the food (in the solo condition), before it was called back into the middle compartment by one of the helpers to start the next trial (if the animals did not perform the relevant action within the 2-minute time span, the trial was considered unsuccessful). If the animal stepped on the correct marker, the human partner was released and followed the same protocol as in Experiment 1 (see Supplementary Information, a–g). If the animal stepped on the marker in front of the empty shift, both markers were pulled up on the fence by the two helpers with the help of a pulley system, thus making it impossible for the animal to step on them again; these trials were considered a ‘failure’. Subsequently, the helpers behind the right and left shifts, as well as the human partner, turned their backs to the animal and did not move anymore until the helper behind the middle shift called the animal back into the compartment after one minute. Finally, if the subject recruited the human partner in the solo condition, the human partner just walked to the table and stood in the middle at a distance of 3 meters without doing anything else (also in this case the trial was considered a ‘failure’).

In case the subject stepped on the marker next to the empty compartment (wrong marker) or did not step on any marker in more than 50% of cooperation trials in one session, one or more ‘additional training sessions’ consisting of 6–8 trials were conducted to remind the animals that if they stepped on the correct marker with a human being in the shifting compartment, the sliding door opened and the human partner entered the enclosure (the apparatus was moved so that it was not in contact with the fence, i.e. non-functional; see Supplementary Material for details). The rationale for these additional training sessions was that we had initially only performed a minimum of training, which allowed animals to learn that the door to the shifting compartment would open when stepping on the marker, but not that a human could then enter the test enclosure, which was a situation the animals had never encountered before. Since we were not primarily interested in whether they understood this association, we introduced these additional training sessions to further ensure that the animals realized how to recruit the human partner. All animals received at least one additional training session, except one dog (Sahibu).

For the analyses, the following behaviours were coded from the videos for each trial: (1) Stepping on the correct marker before visiting the table, (2) Stepping on the correct marker after visiting the table; (3) Table visits: whether or not in each trial the animal visited the table before stepping on a marker (4) Rope-pulls: whether or not the animal pulled the rope before recruiting (stepping on a marker). Behaviours were coded from videos using the Solomon Coder (Solomon Coder beta 17.03.22). Twenty percent of the data were coded by a second rater. Interobserver accordances (calculated with Cohen’s kappa) for the coded behaviours were all above 85%.

Generalized Linear Mixed Models (binomial distribution) were calculated with the number of trials in which each behaviour, as outlined above, occurred as dependent variables, and condition (solo vs. cooperation), session and species as explanatory factors, given the number of trials for the two conditions was not the same, we used the “weight’ function to account for this. A species by condition interaction was included to assess whether wolves and dogs behaved differently in the two conditions. Furthermore, we included whether an animal had received an additional training session prior to the test session (binomial yes-no) as a control factor and included subject identity and human partner identity as random factors.

In all cases a full model with all predictors and random factors was compared to a null model with random and control factors included. All statistical computations were performed with R Studio (Version 1.0.136), package lme4^26^.

## Results and Discussion

**Figure 2:**
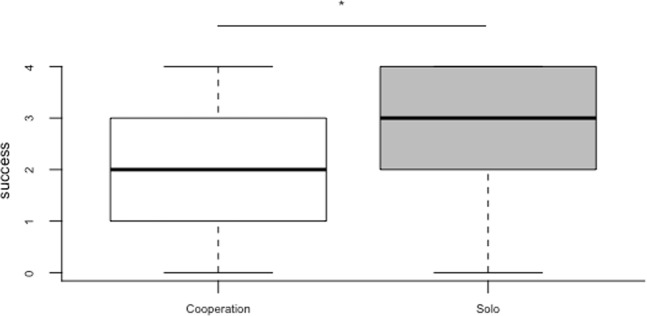
Box plots of the number of trials the animals were successful in the solo and cooperation trials across sessions of experiment 2a. Shaded boxes represent the interquartile range, bold horizontal lines within boxes denote the median, and whiskers indicate the 5th and 95^th^ percentile. Asterisk indicates a significant difference between conditions p < 0.0001.

Overall, animals were successful in 64% of the trials across all sessions and, more specifically, they were successful in 72.5%, solo trials and 56% of cooperation trials (Fig. 2). The full model with condition, session and species differed significantly from the null model with the control and random factor (χ2 = 26.18, p < 0.0001). In line with our prediction, animals were more successful in solo than cooperation trials (χ2 = 24.73, p < 0.0001). We found no species condition interaction (χ2 = 0.05, p < 0.0001), and no evidence for an effect of species (χ2 = 0.0118, p = 0.91) or session (χ2 = 0.86, p = 0.35).

*Stepping on the marker adjacent to the human partner compartment before a table visit* occurred in 23% of total trials (solo and cooperation) (full-null model comparison: χ2 = 1.76, p = 0.62). Instead, in 71% of trials, the animals *first visited the table* (above chance, Binomial test p < 0.001; full- null model comparison (χ2 = 1.5, p = 0.68). Hence regardless of condition, or species, animals first visited the table.

In 57% of trials in the cooperation condition and 72.5% of the trials in the solo condition, the animals *pulled the rope when visiting the table*. All animals pulled the rope in at least one trial in the solo condition and all but one animal pulled it in at least one trial in the cooperation condition. The full model significantly differed from the null model (χ2 = 38.77, p < 0.0001). A species by condition interaction emerged (χ2 = 6.54, p = 0.01). Dogs pulled more in the solo than the cooperation condition (χ2 = 24.02, p < 0.0001) and a session effect emerged (χ2 = 4.9, p = 0.03). In wolves, no condition effect emerged (χ2 = 2.77, p = 0.096), but a session effect was found (χ2 = 3.88, 0.048).

In cooperation trials (n = 361), animals *stepped on any (both correct and incorrect) marker after visiting the table* in 47% of all trials (i.e. at random). Nevertheless, when they did step on a marker, they were more likely to step on the correct (142) than incorrect (28) one (Binomial, p < 0.0001). Stepping only on the marker adjacent to the human partner compartment after visiting the table (so both the correct actions and in the correct sequence) occurred in 39% of all trials. The full model considering this dependent variable significantly differed from the null model (χ2 = 228.7, p < 0.0001). Stepping on the marker adjacent to the human partner compartment after visiting the table was more likely to occur in cooperation trials (n = 142) than solo trials (n = 1) (χ2 = 31.47, p < 0.0001), with no evidence for a condition by species interaction (χ2 = 0.003, p = 0.96), an effect of species (χ2 = 0.43, p = 0.51) or session (χ2 = 0.06, p = 0.81). In contrast, across all 720 trials (solo and cooperative), animals stepped on the marker next to the empty enclosure (regardless of table visit) in a total of 63 trials (8.7%), 20 in solo and 43 times in the cooperation trials.

Considering only cooperation trials, in which the animals first visited the apparatus (n = 259), animals recruited a partner (i.e. stepped on the correct marker) in 52% of trials. Thirteen 13 of the 15 animals were significantly more likely to recruit the partner (by stepping on the marker adjacent to the human partner compartment) in cooperation trials than in solo trials at the individual level (see Table 2).

**Table 2:** Percentage of trials in which animals recruited the partner by stepping on the correct marker, considering only trials in which the animal first visited the table.

Individual	Species	Solo	Cooperation	Individual	Species	Solo	Cooperation
Yukon	wolf	0	42.1**	Nuru	dog	0	65.0***
Chitto	wolf	0	47.1*	Asali	dog	0	61.0***
Nanuk	wolf	6.25	60.0**	Meru	dog	0	63.6***
Aragorn	wolf	0	64.3***	Nia	dog	0	22.2
Kaspar	wolf	0	38.8*	Binti	dog	0	37.5
Tala	wolf	0	42.1*	Sahibu	dog	0	85.7***
Kenai	wolf	0	58.0**	Pepeo	dog	0	62.5**
Geronimo	wolf	0	42.1***				

At the individual level, 13 of 15 animals recruited the partner significantly more often in cooperation than in solo trials (*p < 0.05, **p < 0.01, ***p < 0.001; Fisher exact test).

The results regarding ‘partner-recruitment’ are particularly interesting, because, despite indications that the animals showed some understanding of the task or had learnt to step on a marker to recruit a partner, they nevertheless recruited a partner in only 52% of cooperation trials, in which they had first visited the table (Table 2). One possible explanation for this comparably low performance level is, that for the animals, it was a considerable challenge to leave the table and head all the way back to the start location 25 meters away to step on the marker, due either to limitations in their inhibitory control abilities or in their motivation to pay the cost of travelling back.

## Experiment 2b: Cooperative vs. Solo Condition (Short Distance)

### Methods

To evaluate the possibility that our setup was too challenging because it required moving quite far from the apparatus to recruit the partner, an experimental session with 8 trials (4 cooperative, 4 solo) was added, in which the spatial configuration of the setup was changed so that the ‘marker’ used to open the door and the table were close together rather than being located on opposite sides of the enclosure. We predicted that animals would be more likely to recruit the partner (step on the marker after visiting the table) in the cooperation trials of this new setup than in the final session of the original setup.

We ran a model with the likelihood of stepping on the marker after visiting the table as the dependent variable and session type (original vs. new setup) and species as explanatory factors. We also included condition (solo vs. cooperation) and the interaction between condition and session type, since the change in configuration might have affected the animals’ performance differently, depending on the condition.

## Results and Discussion

**Figure 3:**
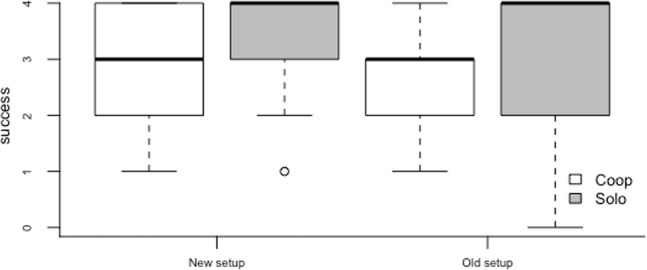
Box plots of the number of trials in which the animal stepped on the marker next to the human compartment after visiting the table in cooperation vs. solo trials, in the last session of the original setup (Old setup) and in the session with the new setup (New setup). Shaded boxes represent the interquartile range, bold horizontal lines within boxes denote the median, and whiskers indicate the 5th and 95^th^ percentile.

Comparing the last session of experiment 2a with the session in which the new setup was presented (2b), we found the full model to be significantly different from the null model (χ2 = 100.2, p < 0.0001). Stepping on the marker next to the human compartment after visiting the table occurred in 70% of trials in the new setup session, which was significantly higher than in the last session of the old setup (58%) (χ2 = 4.06, p = 0.04) and they did so more in cooperation vs. solo trials (χ2 = 33.21, p < 0.0001), but we found no interaction between the two (χ2 = 0.25, p = 0.62) and no effect of species (χ2 = 0.44, p = 0.60; Fig. 3).

Despite the increase of stepping on the correct marker in cooperation trials with this new setup, the overall success rate (in solo and cooperation trials) did not increase (from 70% in the last session with the original setup to 78% success in the new setup; no difference between full model with species, setup and condition as explanatory variables and the null model χ2 = 6.2, p = 0.1). Accordingly, in the final session of the original setup and the session with the novel setup, success rate was no longer significantly affected by the solo vs. cooperation conditions, indicating that 1) both conditions were similarly ‘easy’ for the animals to be solved, and 2) initial inhibition problems of the test subjects were overcome across the original test sessions.

The results of study 2b support the concern that the distance between the marker and the table may have been an issue for the animals: when the marker (and partner) were placed considerably closer to the apparatus, the animals were more likely to step on it and recruit the partner than in the original configuration, suggesting that both their inhibitory control abilities and their motivation were less challenged in this situation. Both our wolves and dogs were tested in an inhibitory control task, where they needed to walk away from a food reward in order to reach it by detouring a fence^20^. While wolves outperformed dogs in that specific task, most animals could manage to walk away from the food at one point during the trial and eventually succeeded. However, the distance to travel was much shorter than in the current task (3 m vs. 25 m).

## General Discussion

Overall, the wolves and dogs adjusted their behaviour to the different versions of the string-pulling task quite well. In the delay condition, they refrained from pulling the rope out and waited for their partner to arrive. In the second experiment, after visually inspecting the apparatus and/or ‘testing’ the rope, they either pulled the rope to solve the task in the solo condition or went to recruit a human partner in the cooperative condition.

While the wolves and dogs did not show much improvement over the sessions, they, like all other species that have been tested in this setup, had extensive experience with other conditions of the string-pulling paradigm beforehand^7–10,12,14,15,25^. In the basic version of the cooperative loose-string paradigm, the animals need to synchronize their behaviours to pull at the same time in order to be successful, otherwise the rope is pulled out by one individual and the task can no longer be solved. Considering that individuals in the dyads are unlikely to always arrive and pull the ropes by chance at exactly the same time, at least some delay is inherent to this version of the task. Longer delays (as tested in the ‘delay condition’) pose an additional challenge to the animals on top of understanding merely the need for a partner. Along these lines, several studies have shown that chimpanzees^7^, elephants^9^, dolphins^11^ and keas^12^ could wait for up to 60 seconds for the partner. However, the procedures adopted to test these delays were based at least partly on an incremental presentation of longer and longer delays, resembling a ‘shaping’ procedure instead of a test of the animals’ understanding. In contrast, animals that failed in the delay condition, (pet dogs with human partners^25^, rooks^15^, African grey parrots^14^ and ravens^12^) were all tested in conditions not including incremental delays but rather set times (e.g. 10–15 seconds). Nevertheless, they probably also experienced some shorter delays in the initial conditions, if their partner did not arrive at exact the same time at the apparatus.

In the current study, wolves and dogs were confronted with a 10 second delay and performed rather well, with most individuals making no more than 2 mistakes across 12 trials. However, our wolves and dogs probably had more experience and training with the string-pulling apparatus than the bird species and pet dogs that were tested with similar methods. For example, dogs in our study performed much better in the delay condition than pet dogs in the study by Ostojić and Clayton (2014)^25^, likely due to the fact that some understanding of the need for the partner was acquired by our animals when they were presented with a two-apparatus condition^24^, requiring coordination with the partner in both space and time. Alternatively, it is possible that the dogs’ success was due to their propensity to stay close to humans. This was indeed observed in the two-apparatus condition of the previous study, where dogs followed the human from one tray to the other rather than initiate the action themselves (^24^; see also^27^). However, such attraction cannot explain the dogs’ behaviour in the second experiment of our study, in which they recruited the human partner only in the cooperative trials.

Differently from dogs, wolves had already been tested in a delay condition with a conspecific^8^, and their success rate in the two studies was similar. Interestingly though, when tested with conspecifics, wolves did not have more experience than the tested birds and pet dogs. Nonetheless, they waited at least 10 seconds for their partner, suggesting that they either learn faster^32^, or that they have a better causal understanding than dogs, as has been previously suggested^29–31^.

We did not find a session effect in the delay condition, which is likely due to their previous experience with the string-pulling apparatus^25^. Experience with the task appears to increase the animals’ understanding of the need for a partner, thus increasing their ability to wait, as was demonstrated in ravens^13^.

In addition to (i) an appropriate pulling inhibition and (ii) the understanding that a partner is needed to help in the cooperative string-pulling task, experiment 2 required animals to discriminate between the solo and cooperative apparatus conditions. The wolves and dogs showed a success rate of 66% overall and 78% in the last session with the new spatial configuration. At the individual level, all eight wolves and five of seven dogs recruited a partner more often in cooperation than in solo trials. The general rule adopted most often seemed to be: approach the table, look, pull a little, stop pulling, and go back to step on the marker to recruit the partner, if it gives way, or keep pulling and get the food, if the rope does not give way. Interestingly, the dogs pulled the rope less often than the wolves in the cooperation trials, suggesting that they might have relied more on the visual inspection of the table or had better inhibitory control than the wolves in this set-up. Both dogs and wolves relied more and more on visual inspection over the trials e.g. they learned to discriminate between the rope configurations or got better in inhibiting their urge to pull the rope. All elements of the rule they applied were likely acquired by the animals during the extensive testing in previous studies, and the training procedure of stepping on the marker to open the door included in this study. It is still remarkable that from the beginning of this experiment they had the capacity to combine these elements and to adjust them flexibly to the task requirements.

Whether or not the animal merely combined the different elements that they had learned during the training procedures to solve the task or whether they indeed showed some understanding of the need of a partner can still be debated. After the animals went to the table and realized that they cannot solve the task by themselves (either by visual inspection or by shortly pulling), they might have approached the marker nearest to the door behind which a human was waiting because of the previous training, where doing so was rewarded with food. Thus, preferring the side with the human might have reflected that humans are associated with food. While this can be a possible explanation of the recruitment behaviour and correct side choice, there are two results that do not support that the animals merely combined the various elements of the training: (1) If indeed the training to step on the star (to open the door) and/or approach the human was overly rewarding, one would have expected that the animals always first go to the star that was much closer to their entry point and had been more recently rewarded than the table. However, in 71% of trials the animals first visited the table. (2) The fact that the animals did not significantly increase their performance across sessions, when the ‘marker-stepping’ behaviour was additionally rewarded, does not support the idea that the animals just followed their trained responses. It hence seems likely that the decision to go back to the marker was contingent on what was found at the table. Accordingly, it is likely that the wolves and dogs had at least a basic understanding of whether or not a partner was needed to solve the given task.

In chimpanzees, the only species tested in a similar setup including active recruitment, seven of nine animals discriminated between the two conditions and recruited a partner in the cooperative trials while refraining to do so in the solo trials^7^. Unfortunately, the behaviour of the animals was not described in that study, so it is unclear how the animals arrived at their decision to recruit a partner (i.e. if they merely inspected the apparatus without trying it out, or if they tried to pull the rope). Similarly, also the other studies using solo vs. cooperative apparatuses^10,14,15^ provided no information on how the animals reached their decision, which prevents determination of the elements used to understand the task, or to which degree trained elements might explain the animals’ performance.

Across the two experiments we found no differences between wolves and dogs. The lack of a species difference is rather surprising given previous results showing that wolves outperform dogs when cooperating with conspecifics^8^ as well as demonstrating better causal understanding^29^ (but see^30^). Accordingly, these results do not support hypotheses proposing that human selection for specific traits lead to increased cooperativeness and socio-cognitive skills in dogs compared to wolves. Such traits include better social skills^33^, increased attention^34^, reduced fear and aggression^35,36^, and hypersociability^37^. Instead, the results suggest, in line with other studies^38,39^, that, when highly socialized in a comparative way and cooperating with familiar human partners, dogs and wolves employ similar cognitive skills (or use a similar set of simple rules) to solve the task. These results thus support the canine cooperation hypothesis, suggesting that the cooperative skills of dogs with humans are based on the cooperative nature of their ancestors, instead of reflecting newly derived skills evolved during domestication^40^.

In conclusion, our results show that both wolves and dogs successfully recruit a partner in a task requiring cooperation, thereby adjusting their behaviour appropriately to whether a partner is needed or not. Although the possibility cannot be fully excluded that the animals followed a simple set of rules rather than understanding the role of the partner, it is nonetheless remarkable that they were able to correctly make decisions in the current set-up, thereby reaching a moderate cooperative success. This is in line with other studies showing that many animal species including dogs often use simple decision rules for problem solving, if these suffice to accomplish a task^41,42^.

## General Materials and Methods

Details of the subjects, testing, training, coding of test and observations are included in the SI Materials and Methods, Movie S1, and Dataset S1. This study was discussed and approved by the institutional Ethics and Animal Welfare Committee at the University of Veterinary Medicine Vienna, in accordance with Good Scientific Practice guidelines and national legislation (Protocol number: ETK-01/04/97/2014 & ETK-09/09/2018).

All humans gave informed consent for participating in the experiments and the people visible in video 1 gave informed consent for the publication of these materials in an online open-access publication.

## Supplementary information

Movie S1

Supplementary Information

Supplementary Data File

## Data Availability

The datasets generated during and/or analysed during the current study are available in the supplementary information.
