# Supplementary material for: Wolves and dogs recruit human partners in the cooperative string-pulling task
Source: Sci Rep. 2019 Nov 26;9:17591. doi: 10.1038/s41598-019-53632-1 (PMC6879616; doi:10.1038/s41598-019-53632-1)
Supplement: Supplementary file 2 — Supplementary Information [file 41598_2019_53632_MOESM2_ESM.docx]

### **Supplementary Information**

**Wolves and dogs recruit human partners in the
cooperative string-pulling task**

Friederike Range^1,2^*+, Alexandra Kassis^3^, Michael Taborsky^3^, Mónica Boada^1,4^, Sarah Marshall-Pescini^1,2^

^1^ Domestication Lab, Konrad Lorenz Institute of Ethology, University of Veterinary Medicine, Vienna, Savoyenstraße 1a, A-1160 Vienna, Austria

^2^ Comparative Cognition, Messerli Research Institute, University of Veterinary Medicine, Vienna, Medical University of Vienna, University of Vienna, Vienna, Austria

^3^ Division of Behavioural Ecology, Institute of Ecology and Evolution, University of Bern, 3032 Hinterkappelen, Switzerland

^4^ Grupo UCM de Psicobiología Social, Evolutiva y Comparada, Departamento de Psicobiología, Facultad de Psicología, Campus de Somosaguas, Universidad Complutense de Madrid, 28223, Madrid, Spain.

+Correspondence:

**Friederike Range** [friederike.range@vetmeduni.ac.at](mailto:friederike.range@vetmeduni.ac.at)

ORCID: 0000-0003-3127-5536

Wolf Science Center, Domestication Lab, Konrad Lorenz Institute of Ethology University of Veterinary Medicine Vienna Savoyenstraße 1a, A-1160 Vienna, Austria

**General methods**

### *Subjects*

All animals participating in the study were raised and kept at the Wolf Science Centre (WSC) in Ernstbrunn, Austria (see Table S1 Supporting material for overview of subjects). The wolves were born in reserves in Canada, the USA or Europe. The dogs were born in animal shelters in Hungary or at the WSC. At the age of 10 days, both wolves and dogs were separated from their mothers and hand-raised in groups. All animals except the last generation were exposed to an extensive socialization period for the first 4 months of their lives, in which they had continuous access to human hand-raisers and some access to (adult) pet dogs. The last generation of dogs was raised by puppy raisers during the day and returned to their mothers for the night. At the age of 5 months, all wolves and dogs were integrated into packs of 2-6 individuals in larger enclosures. In all other respects the wolves and dogs were raised and handled under identical conditions and voluntarily participated in various scientific experiments on a weekly basis.

**Table S1**: Overview of all subject animals participating in the present study. Animals had to be successful in experiment 1 to be allowed to continue to experiment 2.

|  | Animal | Species | Sex | Year of birth | Pack | Experiment participation |
| --- | --- | --- | --- | --- | --- | --- |
| 1 | Kenai | Wolf | m | 2010 | 1 | Experiment 1& 2 |
| 2 | Geronimo | Wolf | m | 2009 | 2 | Experiment 1& 2 |
| 3 | Yukon | Wolf | f | 2009 | 2 | Experiment 1& 2 |
| 4 | Nanuk | Wolf | m | 2009 | 3 | Experiment 1& 2 |
| 5 | Kaspar | Wolf | m | 2008 | 4 | Experiment 1& 2 |
| 6 | Aragorn | Wolf | m | 2008 | 4 | Experiment 1& 2 |
| 7 | Tala | Wolf | f | 2012 | 5 | Experiment 1& 2 |
| 8 | Chitto | Wolf | m | 2012 | 5 | Experiment 1& 2 |
| 9 | Asali | Dog | m | 2010 | 1 | Experiment 1& 2 |
| 10 | Binti | Dog | f | 2010 | 2 | Experiment 1& 2 |
| 11 | Nia | Dog | f | 2011 | 3 | Experiment 1& 2 |
| 12 | Nuru | Dog | m | 2011 | 4 | Experiment 1& 2 |
| 13 | Sahibu | Dog | m | 2014 | 3 | Experiment 1& 2 |
| 14 | Meru | Dog | m | 2010 | 5 | Experiment 1& 2 |
| 15 | Pepeo | Dog | m | 2014 | 4 | Experiment 1& 2 |
| 16 | Panya | Dog | f | 2014 | 4 | Experiment 1 |
| 17 | Amarok | Wolf | m | 2012 | 1 | Experiment 1 |

### *Test setup*

The tests were conducted in two outside test enclosures at the WSC. Both test enclosures were equipped with two shifting systems on opposing sides of the enclosure, each separated into 3 compartments, interconnected to each other and connected with the enclosure by multiple sliding doors. The sliding doors can be opened from outside the enclosures by pulling a chain or bar that is connected to the sliding door. The test enclosures were familiar environments for all subjects. Before each experimental session, the animals were given 5 minutes to explore the test enclosure.

### *Experimental apparatus*

We used a string-pulling apparatus comparable to the ones used in previous studies (e.g.^1-3^). The apparatus consisted of a 1.50 x 0.75 m table, supported by four legs that could be adjusted in height (Figure S1). On top of the table, an additional wooden board with two small wooden food trays (20 cm apart from each other) was installed that could be slid forwards and backwards on the table by a 5.20 m long rope threaded through a pulley-system on the wooden board. Both ends of the rope had to be pulled simultaneously to pull the board forward. If only one end of the rope was pulled, the rope would come loose. The apparatus was placed directly against the fence in such a way that the ends of the rope hung into the test enclosure with an approximate length of 1.20 m, and the food trays could not be reached unless the test subjects moved the board closer to the fence by pulling the two ends of the rope. The starting position of the apparatus was setup before each trial with the wooden board pushed to the back of the table, holding the food trays that were baited with pieces of sausage. If the animals tried to acquire the food by using an alternative strategy (e.g. by pawing), a human experimenter positioned on the outside of the enclosure behind the apparatus prevented the wooden board from moving by pulling two separate strings that were attached to the back of the apparatus. A green tarpaulin was attached to the fence behind the apparatus for the experimenter to hide behind in order not to distract the test subject.


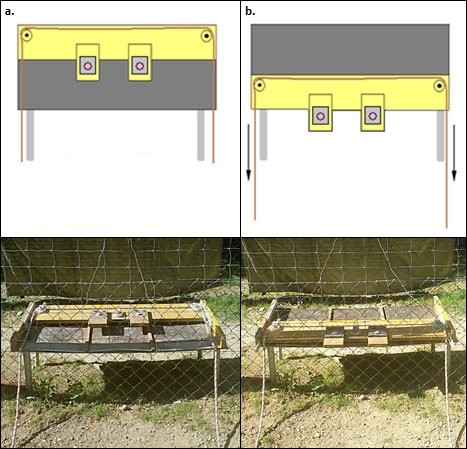


**Figure S1:** The experimental apparatus in the start position (A), with the board positioned all the way back in the `unsolved` position and also in the ‘solved’ position (B), with the board moved forward, the food trays through the fence making them accessible to the test subjects.

### *Experimental procedure*

The tests were conducted in outside test enclosures. After a 5-minute exploration phase, the test subject was shifted into the middle compartment of the shifting system, approximately 40 m distance from the apparatus, where the human partner already waited. The human partner greeted the animal for approximately one minute before she moved either to the adjacent left or right shifting compartment (sequence randomized within and across animals). Then the experimenter entered the test enclosure and prepared the apparatus by looping the rope through the pulley system and placing the remaining rope in to the test enclosure, making sure that both ends were of the same length. She then stepped in front of the apparatus facing the subject and human cooperation partner, called their names and showed them the two rewards (a piece of sausage in each hand; brand “Geiger”, type “Knacker”) to get the subjects’ attention, before placing them on the wooden trays. Afterwards, the experimenter left the test enclosure and the focal subject was released by the helper. The partner was released 10 seconds later. For the detailed procedure and the rules the human partner needed to wait please see supplementary information.

The human partner adhered to the following rules:

a) From the moment the sliding door opened, the human cooperation partner walked directly towards the middle of the apparatus at a normal pace (average speed from the shift to apparatus: 17 seconds). The human cooperation partner did not interact with or look at the animal in any way during a trial.

b) Although approaching the apparatus from the middle, the human cooperation partner selected the side from which the animal’s head was furthest.

c) If the human cooperation partner approached the apparatus first, she chose the animal’s less preferred side (known from a previous study). Following this, the human cooperation partner picked up the rope, held the rope for 10 seconds and then, independently from the animal’s behaviour, slowly and continuously started to pull the rope. If the animal did not approach the apparatus in time or did not take the other rope, the human cooperation partner pulled the rope through the pulley system and the trial ended. However, if the animal approached the apparatus before the 10 seconds of holding the rope had passed, and took the other rope and started to pull, the human cooperation partner started pulling as well. In case the subjects successfully pulled the wooden tray forwards, they received the reward (a piece of sausage) and the trial ended.

d) If the animal approached the apparatus first, the human cooperation partner selected the rope not chosen by the animal. In case the animal did not pull the rope, the human cooperation partner picked up her rope and held it for 10 seconds before she commenced pulling. If the animal pulled the rope by the time the human cooperation partner reached her side of the apparatus, she picked up the other rope (if it was still available) and pulled together with the animal. The trial ended either when the animal pulled the rope through or when the cooperation partners succeeded. In the latter case, both received a food reward.

e) If in situation c) or d) the subjects pulled the rope together and the animal spontaneously stopped and did not start pulling again, the human cooperation partner stopped pulling for 10 seconds, or until the animal restarted, and then kept pulling the rope until it was out. In the case that the animal did not restart pulling, the trial ended.

f) If in situation c) or d) the human cooperation partner chose one rope and the animal then decided to pull the same one, the human cooperation partner continued holding it for a 3 seconds, then released it and took the other rope to avoid any competition and potential conflicts with the animals.

g) If the subjects were successful, it was important that the human cooperation partner got the reward as fast as possible to ensure that the animal did not have a chance to obtain both rewards. If, however, the animal tried to get the reward of the human cooperation partner as well, the human cooperation partner was not allowed to block the animal, but rather stepped aside to avoid any competition and potential conflicts.

h) When a trial ended, the subjects were called back to the shifting compartment by the helper and had the opportunity to greet again (for approximately 1 minute).

**Experiment 2a: Cooperative vs. solo condition**

The aim of the second experiment was to investigate if the wolves and dogs could adjust their behaviour flexibly and recruit a partner depending on whether or not they needed one.

### ***Experimental Setup***

While the loose string-apparatus in the cooperative trials was identical to the one used in Experiment 1, in the solo condition a single rope was attached to a hook in the middle of the apparatus allowing the animals to move the board with the food forward by themselves to gain access to the reward (see Figure S2).

***Recruitment Training***

Using a step-wise training procedure with a secondary reinforcer (clicker), we trained the animals to step on a ‘marker’ (yellow wooden star of approximately 30 cm diameter) placed on the ground, which would result in a person, a helper, - standing outside the enclosure - activating a pulley to open the nearest sliding door. The training was conducted in the test enclosure but in the absence of the string-pulling apparatus. The marker was alternatively placed next to the left or right sliding door with the side counterbalanced across trials, so as to allow animals to acquire the rule that stepping on a marker opened the adjacent compartment. When the animal stood on the marker, the corresponding sliding door opened and the helper threw food into the enclosure. The criterion to be considered fully trained was that animals would step on the marker within 1 minute in 6 out of 6 or 6 out of 8 trials in two consecutive sessions on two separate days.

###

### ***Exposure to the solo condition***

Once the solo apparatus was set- up in the test enclosure and the animal was in the shifting system, the experimenter called the animal and showed the food reward (sausage) to get the subject’s attention before placing the food on the wooden tray. As soon as the experimenter had left the test enclosure, the animal was released from the middle compartment and had 1 minute to solve the trial. The exposure session consisted of a maximum of 8 trials. The criterion to proceed to the test was set at being successful in 6 out of 6 or 8 trials. All animals reached the criterion within 1 session.


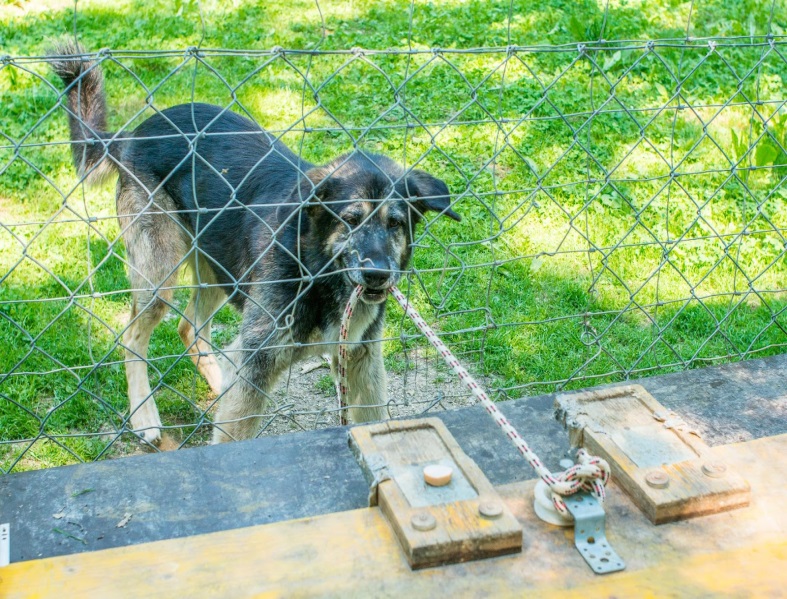


**Figure S2**: In the ‘solo’ version of the apparatus with a rope attached to the middle hook, animals could pull and move the tray forward by themselves.

### ***Additional training sessions***

In case the subject stepped on the marker next to the empty compartment (wrong marker) or did not step on any marker in more than 50% of cooperation trials in one session, one or more ‘additional training sessions’ consisting of 6-8 trials were conducted prior to the next scheduled test session. In these additional training sessions, the animals were, once again, given the experience that if they stepped on the correct marker with a human partner being in the shifting compartment, the sliding door opened and the human partner entered the enclosure (the apparatus was moved so that it was not in contact with the fence, i.e. non-functional; see supplementary material for details). The rationale for these additional training sessions was that we had initially only performed a minimum of training, which allowed animals to learn that the door to the shifting compartment would open when stepping on the marker, but not that a human partner could/would then enter the test enclosure, which was a situation the animals had never encountered before. Since we were not primarily interested in whether they understood this association, we introduced the additional training sessions to further ensure that the animals realized how to recruit the human partner. All animals received at least one additional training session, except one dog (Sahibu).

**Analyses**

In both conditions a trial was considered successful if the subject got access to the food reward by pulling it into reach with the rope, either alone in the solo condition (without prior recruiting), or by operating the apparatus with the human partner in the cooperation condition. Thus, we considered the trial to be a failure when the animals recruited the human cooperation partner in the solo condition, when an animal stepped on the wrong marker, or when an animal pulled the rope out of the pulley system.

**Movie S1:** Solo and cooperative trial of a dog and a wolf.

**References**

1 Melis, A. P. Chimpanzees Recruit the Best Collaborators. *Science* **311**, 1297-1300, doi:10.1126/science.1123007 (2006).

2 Lampe, M., Bräuer, J., Kaminski, J. & Virányi, Z. The effects of domestication and ontogeny on cognition in dogs and wolves. *Sci. Rep.* **7**, 11690, doi:10.1038/s41598-017-12055-6 (2017).

3 Frank, H. Evolution of Canine Information Processing under Conditions of Natural and Artificial Selection. *Z Tierpsychol.* **53**, 389-399, doi:10.1111/j.1439-0310.1980.tb01059.x (1980).
